# An Innovative Model of Positive Education with Traditional Chinese Moral Values: An Evaluation of Project Bridge

**DOI:** 10.3390/ijerph19073797

**Published:** 2022-03-23

**Authors:** Gloria Hongyee Chan, Gabriel Kwun Wa Lee, Chris Yiu Wah Kong, T. Wing Lo

**Affiliations:** Department of Social and Behavioural Sciences, City University of Hong Kong, Hong Kong; glo.c@cityu.edu.hk (G.H.C.); gabriel.lkw@cityu.edu.hk (G.K.W.L.); ssckong@cityu.edu.hk (C.Y.W.K.)

**Keywords:** positive education, moral education, intervention, wellbeing, self, self-cultivation

## Abstract

Project Bridge, as a new, contextualized positive education program, is designed to enhance university students’ character strengths and moral development, resulting in the promotion of their psychological wellbeing. Taking into account the differences between Western and Chinese cultures, the project integrated both Western and Asian concepts and values in the delivery of university education that would likely bring about optimal outcomes. In the evaluation, mixed methods were applied to demonstrate the outcomes of this newly developed positive education program. Pre- and post-test, as well as reflective writing, were adopted to measure the outcomes. Both quantitative and qualitative results demonstrated satisfying outcomes. Implications and future developments are discussed in the conclusion.

## 1. Introduction

The rapid rise of positive psychology has further expanded to school interventions by emphasizing strength enhancement and development for healthier subjective wellbeing, wellbeing, and happier living [1]. The integration of positive psychology into educational paradigms in school settings is referred to as positive education; it attempts to promote students’ wellbeing (interchangeable with happiness), and enhance their academic performance [2,3]. Despite the benefits of positive education [4], positive psychology has been criticized “for refusing to take seriously the moral factors that make life worth living for most people” [3] (p. 87). Its adaptability in different cultural contexts has been questioned [5,6]. For example, some of the character strengths promoted in positive education (e.g., hedonic happiness, self-actualization) are not valued as much in the Chinese context due to the characteristics of the Chinese culture of collectivism, which values relationships and personal obligations [7,8]. Hence, contextualization of positive education in different societies is noteworthy [5] in order to fit with the specific needs of students, and ensure their adaptive, healthy development.

Against this backdrop, a new, contextualized positive education program, Project Bridge, is designed to promote students’ psychological wellbeing by enhancing their character strengths and moral development. In this program, traditional Chinese virtues and moral values have been integrated into the educational framework in order to fit with the developmental needs of students in Hong Kong. Due to the scarcity of evaluation of such contextualized positive education programs, this study seeks to fill the gap by investigating the project’s outcomes, and generating corresponding insights. In the following, the framework of Project Bridge will be illustrated in the light of positive education and its implications when being applied in the Chinese culture.

## 2. Literature Review

### 2.1. Positive Education: Definitions, Features, and Benefits

Positive education is an educational approach characterized by “the application of positive psychology to educational settings” [3] (p. 92). It is based on positive psychology, the study of three dimensions of happiness, namely positive emotions; positive traits (in particular, personal moral virtues and strengths); and positive (enabling) institutions, such as democracy, strong families, and good schools [3] (p. 89) [9] (p. xi). Six core positive traits or character strengths (the “High Six”), including courage, justice, humanity, temperance, wisdom, and transcendence, were argued to be the universal and ubiquitous virtues to reach happiness [3]. Simply put, positive education seeks to cultivate students’ wellbeing in terms of the intrapersonal and interpersonal, “with the concept of happiness at its core” [3] (p. 90).

Based on this premise, positive education can be implemented in different models, such as the Geelong Grammar School Applied Model for Positive Education [2], the PERMA model [10], and the SEARCH framework [4]. The PERMA model is the most common framework for existing positive programs in schools [10]. The PERMA model posits that individuals’ flourishing (i.e., “feeling good” and “functioning well”) [11] (p. 263) [12] can be fostered in the following five domains of wellbeing: positive emotions (P), engagement (E), relationships (R), meaning (M), and accomplishment (A) [9]. Positive emotions refer to “hedonic feelings … feeling joyful, content, and cheerful” [11] (p. 263). Engagement is being psychologically involved and absorbed in activities and life. Positive relationships are the feeling of being integrated into society, and attaining social support from others. Meaning refers to having found the value and purpose of life, and “feeling connected to something greater than oneself” [11] (p. 263). Accomplishment means a sense of competence and achievement [11]. In other words, the PERMA model for positive education seeks to promote wellbeing in terms of students’ hedonistic and eudaimonic happiness [11]. Hedonistic happiness puts more emphasis on short-term positive affections (e.g., pleasure and gratification), whereas eudaimonic happiness requires a flourishing life with a sense of worth and actualization of one’s potential [13].

To achieve its aims and goals, positive education generally involves certain teaching, practices, and activities. Examples include the development of character strengths (e.g., courage, humanity) [6] (p. 300); the learning of social and emotional skills to enhance emotional management and relationship-building [14]; resilience training to enhance students’ ability to cope with stress and solve problems [15]; and mindfulness training to enhance attention to the present moment, and gain awareness of one’s feelings when overwhelmed by anxiety [16]. Benefits of positive education include enhanced self-esteem, self-worth, and life satisfaction, and reduced depression and anxiety, which, in turn, enhance academic performance [4]. For Chinese societies where academic achievement is overly emphasized, which creates stress for students [7], implementing positive education might help cultivate all-round positive development in individuals.

### 2.2. The Applicability of Positive Education in the Chinese Context in Light of Cultural Differences between Western and Chinese Contexts

#### 2.2.1. The Absence of Moral Education in Positive Education

Despite the benefits of positive education, some criticisms have arisen. Positive psychology, which grounds the development of positive education, is scrutinized for over-emphasizing individual hedonic pleasure, and “exclusively” promoting “a particular personality type: a cheerful, outgoing, goal-driven, status-seeking extrovert”, which turns out to be “shallow” [3] (p. 88) [17]. It ignores the moral factors which affect one’s sense of wellbeing and meaningfulness in life [3]. Such moral education is essential for the Chinese context [7]. In Chinese cultures, “whole-person education”, inclusive of “moral, intellectual, physical, social and aesthetic dimensions”, helps cultivate a balanced self with development of “character, mind, body and relationships and the appreciation of beauty” [7] (p. 77). Moral education is greatly emphasized, nurturing an inner personal growth to help attain an ideal society of “xiu shen, qi jia, zhi guo, ping tian xia” (修身、齊家、治國、平天下) [8] (p. 5). The harmonious development of all the aforementioned facets starts with an emphasis on personal obligations, and then develops through an individual’s interactions with family, nation, and the world [7,18]. Seeing the cultural differences between the Chinese and Western contexts in the conceptualization of wellbeing, it is noted that positive education has to consider cultural differences [6] (p. 300). As positive education is built upon Western contexts [6], applying positive education into different countries and districts without contextualization may lead to “underrepresenting the unique experiences of diverse cultural groups” [6] (p. 302) [19,20]. Thus, it is important to have a thorough understanding of the cultural features of a context prior to the application of positive education programs. 

#### 2.2.2. Differences in Cultural Characteristics in Western and Chinese Contexts

Hong Kong, as a former British colony, experiences “dualism” in which Western values and the traditional Chinese Confucian values co-exist [7] (p. 66). The Western and Chinese values contrast, which “may often create conflicts and confusion” [7] (p. 67). Western culture, where individualism is upheld, emphasizes autonomy, individual rights, and personal achievement [7]. Hence, the self is conceptualized “as separate from others” [7] (p. 67), and the self becomes the central part of education [21]. Based on this premise, education is “the learner learning about himself/herself” [22] (p. 150). This learning about self can be differentiated into different aspects in terms of the individual (e.g., the physical self, understanding own strengths and abilities), the social (e.g., one’s relationships with other people), and the self in wider contexts (e.g., awareness of different social roles) [21]. The Chinese culture, where collectivism is upheld, stresses conformity and respect for authorities [7]. Hence, the self is understood in relation to social roles and wider social contexts [23]: “the worth of the individual does not lie in its own right but as a function of its relation to the greater collective to which it belongs” [7] (p. 67) [24]. Understanding the different concepts of self in the Western and Chinese cultures, the meaning of self-esteem is different [25]. Whereas it is characterized by explicit positive self-regard in Western cultures, self-esteem is constrained by the norm of being modest in Chinese cultures [25]. As said in a traditional Chinese proverb, “haughtiness invites loss while modesty brings benefits”, and upholding modesty is to “facilitate social harmony and avert strife” [25] (p. 60).

The different cultures in the Western and Chinese contexts, in turn, constitute differences in morality. Although “all moral codes encompass personal rights, personal duties, and social goals” [26,27], the priorities given to these three dimensions are different between the two contexts. In Western areas, higher priority is given to personal rights than to duties and social goals [26]. Every individual has innated fundamental and equal autonomy and human rights [26]. Therefore, individuals are encouraged to fight for the rights of all people to the highest level of freedom [28]. Aligned to the classification of six moral virtues (i.e., wisdom and knowledge, courage, humanity, justice, temperance, and transcendence) and the corresponding 24 character strengths (e.g., creativity, curiosity, authenticity, kindness, fairness, modesty, and humor), as formulated by Peterson and Seligman [29] (Ch. 2–3), treating people fairly and equally is viewed as a form of the “civic strengths that underlie healthy community life” [30] (p. 412). With the help of virtues and strengths, one is able to lead a life with satisfaction and meaning [31]. Any systems hampering the development of human rights and needs will have to change [26].

In contrast, in the Chinese context, morality is based on “maintaining social order”, in which “role relationships within one’s primary group are especially unchangeable through one’s own efforts” [26] (p. 808). Rather than striving for the human rights of individuals, fulfilling obligations is more important for the sake of pursuing the functioning of society [26]. Respect for authority (e.g., parents and the elderly) and performing appropriate roles (e.g., as a son or a husband) in different relationships (“five cardinal relationships”) is particularly important [7] (p. 82). Investing in relationships with trust and reciprocity is even the norm for people to undergo “the exchange of favors and resources” (Guan xi) [32] (p. 149). As such, moral virtues and characteristics of benevolence (“ren”), obedience, and being “societally oriented” (minimizing “xiao wo” for “da wo”) [7] [26] (p. 808) [33] (p. 3) are valued. It is more important for the individual “to fit into the system rather than for the system to suit the needs” of the individual [7] (p. 73). Living in such a culture emphasizing personal obligations and harmony, people tend not to express inner feelings overtly, and perseverance is highly valued [7]. Making a continuous effort is seen as key to the learning and practice of perfection and excellence, rather than ability (e.g., creativity) [7]. Meanwhile, self-efficacy, self-actualization, and individual hedonistic emotions are less emphasized in the Chinese culture [8].

**Figure 1:**
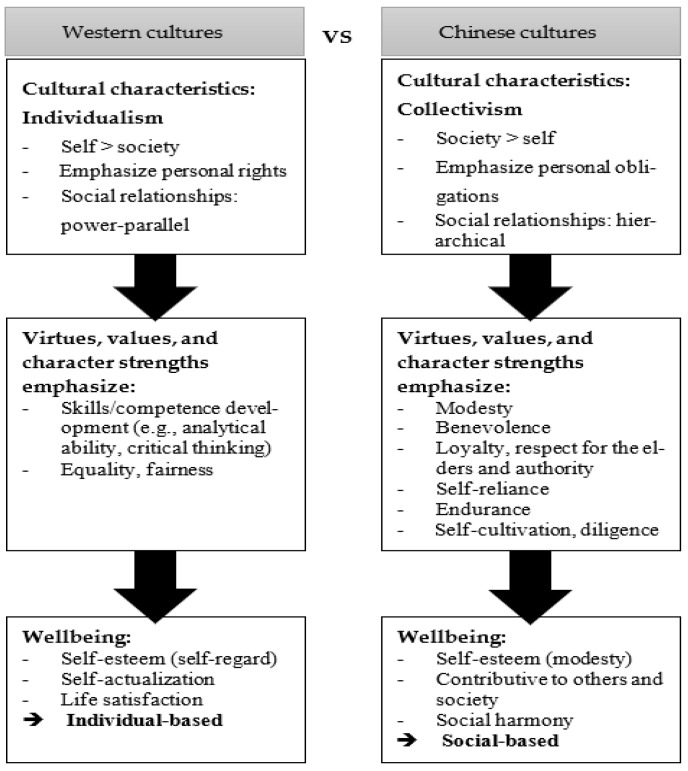
Differences between Western and Chinese cultures which constitute the different kinds of personal education.

In summary, Western-based positive education has to bear in mind the cultural differences when applying it to Chinese contexts because the Western and Chinese cultures uphold conflicting sets of values and moral codes. This constitutes a different understanding of wellbeing between the two contexts. In terms of the development of self, whereas individual hedonistic happiness and competence development (e.g., analytical ability, critical thinking) are valued in Western cultures as ways to achieve wellbeing (self-esteem and life satisfaction), virtues and character strengths based on individual obligations (e.g., self-reliance, endurance) and social harmony (e.g., modesty, benevolence, loyalty, respect) are more valued in Chinese cultures [7,8]. In terms of the development of social relationships, whereas social relationships are built upon equality in Western cultures, social relationships in Chinese cultures are hierarchical (see Figure 1). These constitute the difference in achieving wellbeing between the two cultural contexts. Hence, in order to make positive education more adaptable to the Chinese culture, it is necessary to contextualize the education [5]. The integration of Western and Chinese concepts, values, and skills in the delivery of education would likely bring about optimal outcomes [7], such as extending the Western concept of the self with regard to various social relations. When implementing positive education in the Chinese context, it is necessary to emphasize the development of self in terms of Chinese virtues and character strengths, as well as the self in relation to society, so that individuals are able to pursue wellbeing in Chinese societies.

### 2.3. Insights from the Literature: Formulation of the Project Bridge Framework

#### 2.3.1. Program Aims and Education Components

Given the benefits of positive education for students, and the consideration of Chinese cultures, Project Bridge was implemented in a university in Hong Kong to offer positive education for university students. Project Bridge is a positive education program that aims to build and enhance students’ character, ethics, and morality. To achieve this aim, the positive education framework incorporated the Chinese values and virtues of “xiu shen, qi jia, zhi guo, ping tian xia” (in Chinese, 修身、齊家、治國、平天下), translating into English as: “the ancients who wished to illustrate illustrious virtue throughout the kingdom, first ordered well their states. Wishing to order well their states, they first regulated their families. Wishing to regulate their families, they first cultivated themselves” [8] (p. 5); and “respect work and enjoy company” (敬業樂群) [34], signifying the importance of self-cultivation and self in relation to society. 

**Figure 2:**
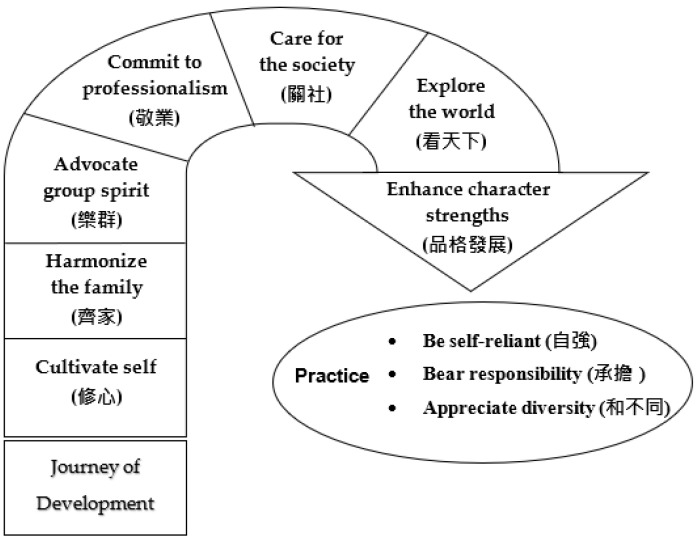
The project framework.

Hence, six domains of self-development are encompassed in this project: (1) cultivating self (修心); (2) harmonizing the family (齊家); (3) advocating group spirit (樂群); (4) committing to professionalism (敬業); (5) caring for society (關社); and (6) exploring the world (看天下) (see Figure 2). These six domains of self-development are nurtured as follows: (1) to help students achieve an understanding of self, and cultivate various individual character strengths (e.g., individual self, self in various social groups and institutions such as family and workplace); (2) to help students extend the development of self into different social systems, bridge them with different social systems (i.e., from the individual level to the societal/community level), maintain harmonious relationships with diversified social groups and institutions, and understand diversities (e.g., gender, age, ethnic, regional, class, political, and religion); and (3) to help students make better use of their potential to deal with life challenges, contribute to society, and attain a balance between actualization of self-identity and the social self so as to achieve better functioning in life. Simply put, the development of these six domains is related to enhancement of the self in terms of inner strengths, morality, and social relationships under traditional Chinese ethics [35,36,37]. This aligns with the eudaimonic dimension of positive education, which seeks to achieve flourishing in terms of actualization of self-potential, and maintenance of social relationships [3]. With these qualities, the expected outcome will be the achievement of wellbeing in terms of the following three daily practices: (1) being self-reliant (自強); (2) bearing responsibility (承擔); and (3) appreciating diversity (和不同), which is in line with the concept of wellbeing in Chinese contexts, as stated in the above literature [7,8,26] (see Figure 2). 

#### 2.3.2. Theoretical Foundations for the Program

**Figure 3:**
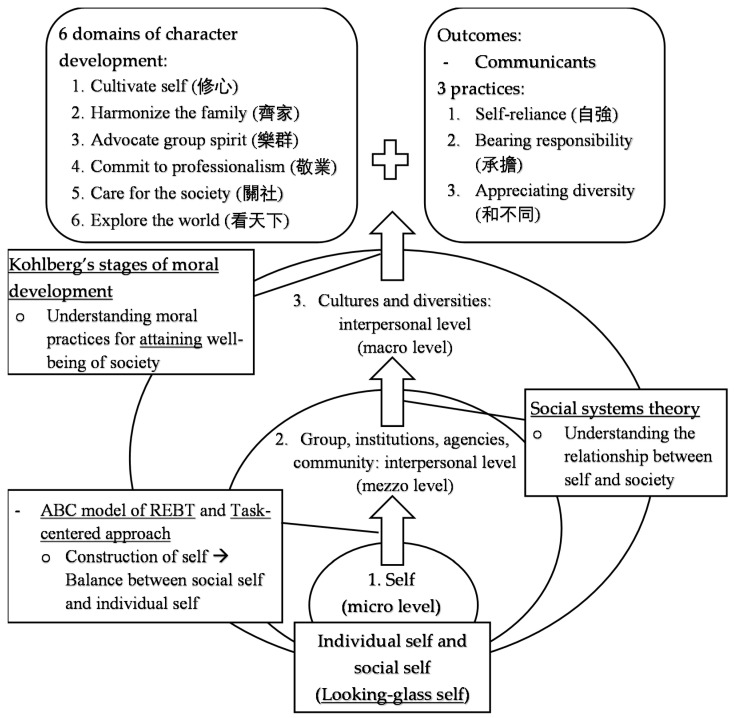
Application of the theoretical concepts and expected outcomes of project framework.

To achieve the expected educational outcomes, the following theoretical concepts were included, with the use of a wide range of educational tools, including lectures, classroom activities, group discussions, and online learning platforms, to implement the education (see Figure 3).

1. Theories of self: The understanding of self comprises both the personal level—i.e., one’s overall perception of oneself in terms of competence and other attributes [38]—and the interpersonal level, i.e., the self derived from one’s interactions with other people [39]. Cooley [39], who put forward the concept of the looking-glass self, emphasized the link between the individual and society. He believed that the self could not be understood as existing independently of the social environment and others’ perceptions of oneself. The self is not merely an individual understanding of the self-concept, but a product of social interactions and evaluation by significant others [40]. These theoretical concepts of self are helpful for students to understand self in terms of actual self and social self, and the differences between the two.
2. Social systems theory: The relationship between self and society can be illustrated by social systems theory [41,42]. Social systems theory posits that society is formed by multifarious social systems (e.g., family, school, organization, and culture). Individuals, groups of individuals, and other social units are interdependently related, and influence each other in a functional manner [41,42]. By understanding the relationships among self, others, and the environment [43], individuals can: (1) understand the diversities of self, and extend the understanding of self to social roles and social identities; and (2) further understand how their self-concepts are influenced by the environment and significant others, developing a broader perspective of looking at themselves and their problems, and thereby making better use of the self (e.g., inner strengths and resources) to contribute to society, and cope with challenges in life.
3. The ABC model of rational emotive behavioral therapy (REBT) and task-centered approach: Apart from the above, two action approaches in daily social interactions were taught. Firstly, the ABC concepts of rational emotive behavior therapy (REBT) are introduced. REBT is a therapy aimed at helping individuals change their irrational beliefs to reduce their negative emotions, and enhance their sense of wellbeing [44]. Ellis [44] developed an ABC model in which A, B, and C stand for activating event, belief, and consequence, respectively, based on the premise that individuals’ emotions are rooted in their interpretation of events. In the model, it is posited that an external event (A) does not necessarily arouse emotions (C), but beliefs (B), particularly irrational ones, do [45]. Hence, this model can help individuals identify their irrational beliefs, and undergo cognitive restructuring in order to reduce emotional problems and achieve healthy, positive consequences. Secondly, the project teaches students the task-centered approach [46,47], which is a short-term social practice adopting a problem-solving orientation. It is posited that problems arise from imbalances with the environment, and individuals are active problem-solvers to deal with such imbalances. Hence, this approach aims to assist people in reviewing their values and beliefs, and solving target problems in their daily lives using adaptive coping strategies, through which they learn problem-solving strategies and enhance self-efficacy. These two approaches are useful for students’ construction of self, helping them establish a positive self and achieve a sense of wellbeing by striking a balance between the social self and the actualized self. In other words, individuals can achieve a self-preferred identity while being able to fulfil societal expectations and solve daily problems.
4. Kohlberg’s stages of moral development: Kohlberg [48] put forward three levels of moral development (pre-conventional, conventional, and post-conventional), and the corresponding six stages of moral development. In each stage, moral thinking and judgment are undertaken with different orientations, from avoiding punishment and self interest-based (i.e., pre-conventional level), maintaining social relationships and social order (i.e., conventional level), to the pursuit of individual rights and social justice (i.e., post-conventional level). It is posited that “all individuals in all cultures use the same basic moral categories, concepts, and principles, and all individuals in all cultures go through the same order on a sequence of gross stage development, though they vary in rate and the terminal point of development” [49] (p. 175). Including the traditional Chinese values and moral codes in the program, it seeks to overcome the limitations of the theory of “Western ethnocentrism” [26] (p. 806), and addresses the Chinese ethnics of “independence” and “social harmony” more [33] (p. 2). In other words, they could become communicants, which is defined in this paper as people who: (1) have a good will (i.e., morally good intentions); (2) have the ethics of care and moral conscience; and (3) respect diversity, and listen to all voices [50,51,52] (i.e., have reached stage six of moral development while endorsing the Chinese virtues of seeking social harmony). With the understanding that the nature of communication problems is relevant to the “sense of self and in relation to otherness” [50] (p. 255) [53], it is important for individuals to cultivate and endorse moral conscience and ethical values, addressing differences, awareness of power relations, care, and respect for all others’ autonomy and feelings, as well as social responsibility, so as to maintain good social relationships and citizenship [50,51,54]. Simply put, being a communicant is coherent with the three practices of the project framework for developing character strengths, and ensuring the morality of the individual, which is in line with the spirit of positive education.

In sum, the above theories aim to help enhance students’ understanding of self in different dimensions, as well as the understanding of self in relation to society. As students understand how their sense of self is formed, they will be able to recognize their inner strengths and resources on a deeper level and from a broader perspective, without being influenced simply by how other people in society see them. As such, they are able to achieve a balanced self and all-round wellbeing, recognizing their own abilities while being able to handle their relationships with society, and cope with various challenges in life. Also, it is expected that they will become socially responsible persons and communicants who have good will, and show ethics of care and moral conscience for others in society.

#### 2.3.3. Content of Project Bridge

Thirteen lectures were arranged for students in each semester to enhance their ethical and philosophical thinking and promote character education for youths in tertiary education. The first three lectures cover the theories of self and social systems theory to acquire basic knowledge about youth studies, revealing their individual self and social self for later discussions in the course. Then, the ABC model and task-centered approach will be taught to uncover the impacts of irrational beliefs on various social contexts, resulting in a better balance between social self and individual self. After the theoretical knowledge, real-life youth issues in contemporary society, such as LGBT+, cybercrimes, bullying, drugs, conflict management, slash youth, and positive youth development, are discussed to connect the social issues with the learned theories, contextualizing the former learnings with various settings. Particularly, Chinese moral values are merged into the curriculum through discussion and demonstration in lectures. Examples used in the lectures were updated from time to time to match with the social situation that students were experiencing.

Besides, students are engaged in discussions and debates after each lecture to connect their learning with current issues related to youth development in the local and global contexts. Through reviewing different perspectives among group members, students can deepen their understanding, and integrate their experience with concepts learnt in the lectures. They have to collaborate and build upon each other’s opinions to reach a consensus. In this process, effective communication and interpretation skills are required to produce the final outcome of their discussion, giving rise to reciprocal interactions among students.

Apart from the lectures and in-class activities, tutors will arrange individual consultation sessions to help students develop a deep view of youth issues, and help them reach an appropriate academic standard throughout the preparation of course assignments. The interaction between instructors and students is meaningful to clarify theoretical understandings, enhance learning outcomes, and transmit positive values throughout the preparation of, and consultations on, assignments. 

### 2.4. Present Study

Due to the scarcity of evaluation of contextualized positive education programs in Chinese contexts, this study aims to investigate the outcomes of Project Bridge, a university course, in terms of students’ self-development and development of social relationships. It is expected that corresponding insights for implementing positive education in Chinese contexts will be generated.

## 3. Method

Since the project is operated as a university course, all students enrolled in the course are eligible for participating in the evaluation on a voluntary basis. Purposive sampling method is adopted.

### 3.1. Quantitative Method

Questionnaires with a pre-test and post-test design were administered to students to measure if there were any significant changes in students’ self and the three practices, as shown in Figure 2. Pre-test and post-test questionnaires were distributed online to students before the first session and after the last session through The Question Pro Research Edition, and completed in a self-administered manner. Items were displayed in both Chinese and English. Back translations by two independent translators were applied to those scales without a published Chinese version (i.e., Collective Self-Esteem Scale and Emotional Maturity Scale).

#### 3.1.1. Measurement or Instrument

The following items and scales were included in the questionnaire.

##### Demographic Variables

Students’ demographic backgrounds were sought, including gender, age, academic program studied, and monthly household income.

##### Self Variables

(1) Rosenberg Self-Esteem Scale [55]. This ten-item scale helps assess students’ level of self-esteem before and after attending the program. It adopts a four-point Likert-type scale (1 = strongly disagree; 4 = strongly agree). Items, such as “On the whole, I am satisfied with myself.” and “I take positive attitude towards myself.”, are used to measure the perceived self-esteem of participants.
(2) Moral Development Scale for Professionals (MDSP) [56]. This 12-item scale helps assess students’ endorsement of moral and ethical values before and after attending the program. It adopts a five-point Likert-type scale (1 = do not agree at all; 5 = agree completely). Items for stage 3 (e.g., “A necessary condition for an action to be good is a good thought behind.”), stage 4 (e.g., “Immoral conduct breaks established laws and rules.”), and stage 5 and 6 (e.g., “Usually possible to reach consensus in moral issues.”) are covered in the scale.
(3) Collective Self-Esteem Scale (CSES) [57]. This 12-item scale helps assess students’ level of self-esteem in terms of group membership, which reflects students’ relationship with others/social groups before and after attending the program. Examples of the items are: “I am a worthy member of the social groups I belong to.” and “Overall, my social groups are considered good by others.” It adopts a seven-point Likert-type scale (1 = strongly disagree; 7 = strongly agree).
(4) Emotional Maturity Scale (EMS) [58]. This 48-item scale helps assess students’ level of emotional maturity before and after attending the program. For example, “Do you feel that you are short-tempered?” and “Do you lose your mental balance (poise)?”. It adopts a five-point Likert-type scale (1 = never; 5 = always).

##### Being Self-Reliant

(1) Personal–Social Development Self-Efficacy Inventory [59]. A total of 44 items were applied to assess students’ level of self-efficacy before and after attending the program. It adopts a six-point Likert-type scale (1 = very unconfident; 6 = very confident). The scale covers various aspects, including understanding self (e.g., “make use of my strengths and improve my weaknesses”), understanding others (e.g., “stand in others’ shoes and consider their feelings”), marriage and family responsibilities (e.g., “think over the relations between children and marriage when I prepare myself to be a parent”), handling setbacks (e.g., “master problem-solving skills in face of problems”), health and physical training (e.g., “develop a balanced eating habit”), cooperation and team spirit (e.g., “handle disputes in the process of cooperation when participating in activities.”), leadership skills (e.g., “organize and plan activities”), management of stress and emotion (“control my emotions”), self-evaluation skills (e.g., “obtain learning opportunities in the process of self-evaluation”), thinking and expression skills (e.g., “reflect my opinion using different ways”), and personal goal setting (e.g., “plan how to attain the different goals in life”).
(2) Empowerment Scale [60]. This 28-item scale, including items such as “I feel I am a person of worth, at least on an equal basis with others.” and “People have a right to make their own decisions, even if they are bad ones.”, helps assess students’ level of empowerment before and after attending the program. It adopts a four-point Likert-type scale (1 = strongly disagree; 4 = strongly agree).

##### Bearing Responsibility

(1) Personal Responsibility Scale for Adolescents [61]. This 23-item scale, including items such as “I am aware of how my behavior impacts on other people.” and “I choose how to respond in situations.”, helps assess students’ sense of responsibility before and after attending the program. It adopts a four-point Likert-type scale (1 = strongly disagree; 4 = strongly agree).

##### Appreciating Diversity

(1) Openness to Diversity Scale [62]. This 12-item scale helps assess students’ acceptance of diversity before and after attending the program, for example, “I travel to different places to find the uniqueness of that place.” and “I am comfortable working with people whose gender is different from mine.” It adopts a five-point Likert-type scale (1 = strongly disagree; 5 = strongly agree).

#### 3.1.2. Analysis Strategy

The pre- and post-test data were inputted to SPSS 26 to conduct paired t-tests to examine whether students had experienced significant changes in relevant aspects. 

### 3.2. Qualitative Method

The project framework comprises six domains and three practices (Figure 2). Given that the three practices were examined by a quantitative evaluation method, the development of the six domains was examined by a qualitative method: students’ self-reflection. 

According to Fullana and colleagues [63], reflective practice improves students’ self-understanding, and helps demonstrate learning outcomes. In the curriculum, students are required to write a short reflection paper to state their learning outcomes, self-reflections, inspirations, and any thoughts triggered by the lecture. The structure and format are not restricted: students are free to express their learning outcomes in this assignment. Meanwhile, students are encouraged to connect the learning with their personal experiences. This practice is conducted through a written narrative method, which can demonstrate a similar or deeper level of information, structure, and meaning than the oral narrative interview [64,65]. Hence, the students’ self-reflection is adopted as qualitative data to assess the learning outcomes, and evaluate the development in domains.

Qualitative analysis of the reflective content from students can further complement the quantitative data to produce a more comprehensive picture of the learning outcomes by offering detailed explanations in each domain. Hence, the experiences and knowledge that the students have acquired and enhanced by attending the project are revealed.

#### Analysis Strategy

The reflective contents were coded according to the six domains shown in Figure 2 to offer detailed descriptions of students’ development. In order to identify the categories, keywords, such as personal, self, parents, family, friend, peer, career, pathway, society, marginalization, world, and country, were applied to locate relevant content in the reflection papers. Extensions of the keywords (e.g., myself, personality, etc.) were also screened during identification. Some words that commonly appeared in the personal reflections were also listed in the keywords to identify subsequent reflections (e.g., conflict, argue, LGBT+, etc.). With the keywords applied, relevant quotations were extracted.

Although many students mentioned the keywords, and made some reflections on the domains, only in-depth reflections were counted to ensure that the content reflected the growth of students. Hence, the context of reflection was assessed to check the depth. Reflections that contained solely descriptive and repetitive content of the knowledge, theory, or course materials were excluded. Some students also demonstrated their development in the framework domains by reflecting on their past experiences before attending this course (e.g., employment and school activities), but these reflections were excluded. 

Meanwhile, if one student made multiple comments in one domain, all quotations were extracted for qualitative analysis, but the student was only counted as one participant in the results to illustrate the number of students inspired in each domain. 

## 4. Results

Project Bridge reached 950 undergraduate students throughout its implementation across four semesters at City University of Hong Kong (Table 1). The majority of students (71.4%) were aged 20–22, and 1.6% were aged 25 or above. The most common monthly family income for students (46.3%) was $10,001–$29,999, whereas $30,000–$49,999 was ranked the second (25.6%).

### 4.1. Results of Quantitative Evaluation

Cronbach’s alpha for each scale varied between 0.63 and 0.968, which were acceptable (Table 2). Given that the number of female participants was near double that of male participants, a chi-squared test was also run to check whether gender produces any influence on the outcomes. It was found that gender did not produce a significant impact on the overall program evaluation.

The data were analyzed with paired t-tests that compared the scores before and after the students attended the project. We found significant changes in all evaluation items: students’ self-esteem, personal–social development, self-efficacy, empowerment, collective self-esteem, emotional maturity, personal responsibility, openness to diversity, and moral development. The results demonstrated a satisfactory outcome for Project Bridge, especially its effectiveness in strengthening self-reliance, bearing responsibility, and appreciating diversity (see Table 3). In particular, moral development demonstrated a significant increase in stages 5 and 6. According to the moral development theory [56], individuals reach the upper moral level after passing through lower moral stages. Stages 3 and 4 belong to level 2 morality, whereas stages 5 and 6 are level 3. Therefore, a significant increase in stages 5 and 6 of moral development indicates that a significant number of individuals have already passed through level 2 morality, thus resulting in the insignificance of stages 3 and 4.

To conclude, Project Bridge demonstrated a satisfying outcome. Students’ self-concept and image, morality, and character strengths were significantly enhanced, as reflected through the three practices of being self-reliant, bearing responsibility, and appreciating diversity. Hence, Project Bridge has undoubtedly achieved the intended student learning outcomes, implying the potential of merging Western and Chinese concepts in positive education.

### 4.2. Results of Qualitative Evaluation

Consequently, the qualitative results confirmed the project design, which began with the first domain: cultivating the students’ self, and then continuing to develop the other five domains. Undoubtedly, most students reflected on their cultivation of self, and demonstrated enhanced self-understanding. The other five domains were also demonstrated in a sufficient number of reflections, illustrating students’ development (Table 4). Hence, it can be inferred that students experienced development in all the domains along the journey of development.

Overall, most students made in-depth reflections on cultivating the self after attending the course. Caring for society and advocating group spirit were the second and third leading domains. Next, the domains of committing to professionalism and harmonizing the family ranked fourth and fifth, respectively. Exploring the world was consistently low across the semesters. Explanations in each domain are discussed further below.

#### 4.2.1. Cultivating the Self

For the cultivation of self, most of the students could identify their irrational beliefs after learning the ABC model, which facilitated better self-understanding to deal with irrational beliefs and negative emotions.

- “I could deal with the negative feelings and no longer treat myself as a ‘loser’.”
- “Identifying my irrational beliefs behind the response is the first step for self-change. … Thus, I can think clearly and respond appropriately to the event.”
- “I realized that I should not let all my negative thoughts dominate my mind. After knowing this concept, I feel better about myself, and I start not blaming myself when facing problems during the day.”

By taking an alternative perspective, students built a better and fairer judgment of themselves, and thus, understood the two sides of the coin. This improvement allowed them to be more confident in developing self-reliance, and exploring new potential. With the cultivation of self, students were thus able to react more fairly and objectively when interacting with others.

- “I plan to actively participate in more co-curricular activities in the future to expand my social network and explore my potential. I hope this will allow me to understand my strengths and weaknesses better and particularly enable me to embrace my flaws and imperfections.”
- “It is impossible to fully understand ‘who I am’ and ‘what is self’ by relying on individuals. Sometimes communicating with others is a good way to know your self.”

Hence, through the cultivation of self, they can better understand not only themselves, but also others, resulting in enhancement in other domains.

- “This course has helped me broaden my perspective on other people and myself…It made me reflect a lot on choices that I have made or my family made for me.”
- “To a friend who has gone through that developmental journey, it is beneficial that we express our care and concern, empathy, and regard him positively.”

The reflections confirm that the project design that presumes better self-understanding can generate positive influences in other domains. 

#### 4.2.2. Caring for Society

Regarding caring for society, two guest lecturers were invited to share the issues of LGBT+ and refugees. In the summer term 2019/2020, a transgender person shared his personal experiences, struggles, and public attitudes toward this population. This real story inspired lots of students to obtain a deeper understanding of this specific social issue.

- “I realized that transgender people face many challenges in their family relationships, friendships, or even in their daily lives.”
- “I learned more about transgender and how they think they are … They must suffer from pain and take risks to have such major surgery.”

On the other hand, a guest lecturer highlighted refugee issues in Hong Kong in Semester A 2020/2021. The guest lecturer caught a lot of our students’ attention by illustrating the reasons for becoming a refugee, social policy, and the lived hardships.

- “In the past, I seldom glanced at the issue of refugees in Hong Kong as it is not close to my personal interest.”
- “Before attending the class, I would describe myself as a discriminator against those refugees.”
- “Living with a legal identity seems like an ordinary thing to us, but it is hard to imagine there is still a group of people who live in Hong Kong without an identity card.”

In the reflections, students indicated a vast contrast in their understanding of this social issue and population before and after the lecture; hence, more reflections were triggered regarding their care for society. The project also covered other social issues, such as youth suicide, bullying, drug abuse, mental health, discrimination, and NEET (Not in Education, Employment, or Training), so the scope and range of students’ reflections were expanded.

#### 4.2.3. Advocating Group Spirit

Peers are a critical source of influence and relationships during adolescence, even outweighing familial and parental influences [66]. According to the reflections, the students became more active in initiating social interactions, more considerate, and more able to appreciate diversity.

- “I also know how to take the initiative, give opinions, dare to communicate with others.”
- “The lecture reminds me not to have a self-serving bias and to be more considerate. … I would consider other people’s thoughts more.”
- “We all need to consider each other as humans and that even the slightest thing can harm someone’s mental health. We all need to respect differences with an open mind… We all need to be more empathetic so that things can get better in life.”

With the above characteristics, students are capable of ensuring smooth and effective group interactions with others in any environment. Moreover, most students gained an understanding of the issue of bullying, since they had experienced or witnessed traumatic bullying experiences in secondary school. In-depth reflections on bullying were cultivated during the lecture on the subject. Some of the students were somehow trapped by their past.

- “What I regret most is when I know my friend became depressed after being bullied, I did not give her any consolation, and I just ignored her feelings.”

Reflective writing allowed students to alleviate past pain, which can be regarded as a self-healing process [67]. Furthermore, some students were able to redefine the trauma with the learned theory, and gain positive development.

- “I was a victim of cyberbullying … What I would do is to be objective and try to respond with rational beliefs … It is not that terrible because I’ve got close friends and family who love me and support me all the time.”

Hence, they can move on and overcome the negative influences in the past to develop self-reliance and healthy relationships with others. 

Development in advocating group spirit also benefits other domains, including harmonizing family, committing to professionalism, caring for society, and exploring the world.

- “I understand the importance of connection in all relationships. I should be more able to share my feelings with my parents and listen to their perspectives rather than refusing to speak up.”
- “Maybe we will have conflicts with our colleagues or supervisors, arguing from our different positions in a job. If we can settle them rationally, the arguments will bring about a positive result, career satisfaction, or stable relationships with each other.”
- “We have to respect and accept the truth that everyone is unique, and we should embrace the differences in our society.”

Group spirit is not restricted to peers, as students can use the learning in their interactions with family members, work colleagues, and members of society. 

#### 4.2.4. Committing to Professionalism

Entering the labor market is the next stage of life for undergraduate students. Committing to professionalism also urged them to think deeply about their future career and life. Some students developed a higher commitment to their future profession. They believed that this course provided meaningful tools, knowledge, and attitudes for them to tackle future challenges in the workplace.

- “The task-centered approach is a good way for me to tackle problems. After learning this approach from class, it is easier for me to solve problems when facing them at university and in my career.”
- “I felt extremely bad before attending the course, as I could not tolerate being unemployed and having nothing to do after graduation. However, the introduction of the ABC model made me reconsider my interpretation of this situation. I started to generate a new interpretation that it does not greatly matter whether I can find a job immediately after graduation, and I can use the free time to learn some new stuff and equip myself further.”
- “I think the current difficulties I face can enhance my resilience, and some low-paid jobs can also cultivate my work skills, increase my competitiveness in the job market, and help me find high-tech jobs in the future … Regardless of job title or status, I should treat my job with respect and rigor, try my best to complete my work, and improve my vocational skills.”

Apart from that, it is probable that due to the relevance of the content, students who wanted to be professional educators deepened their career orientation.

- “I aspire to be a teacher, and all the theory and knowledge in this course equipped me to understand how I can better support my students in the future.”
- “In the future, to prepare me to become a teacher, my goal is to enhance my character strengths, in which the wisdom and knowledge, self-discipline, and humanity that I learned in this course are especially important.”

The students became more dedicated to their study major, and intended to use the learning outcomes of this course to help their future careers. 

With reference to the reflections, students were often bounded by the social expectations of finding a high-paid job without considering their own interests and abilities.

- “We are told by society what is best, like getting into a university, getting a well-paid job, and buying a flat. Profession and money are closely linked. We may have neglected our dreams that might be perceived as cheap or unrealistic, like being an athlete or an actor.”

Through attending the course, students were given an opportunity to rethink and listen to their authentic voices to clarify their future career path, rather than blindly following others’ voices.

- “I believe every individual should have their own definition; they do not need to blindly follow what the general public believes to be good. … I follow my own interest in studying linguistics and want to be a teacher after graduation. Therefore, there is always more than one path to take.”
- “I understand other people’s perceptions of me constitute a great part of my self … However, I am more aware of the phase ‘I’ now … I really want to pursue my dream job as a freelance photographer. The choice between a stable job and a dream job is hard to make. I believe we should try to strike a balance between what we want and what others want us to do.”

Other people’s expectations of our students forbid them from chasing their ideal profession, resulting in the suppression of their career orientation. Building better self-understanding facilitates students to value their thoughts and autonomy, and voice their desired future.

#### 4.2.5. Harmonizing the Family

Family conflicts commonly happen during adolescence, as this developmental stage is a time of irrationality, moodiness, and turbulence [68]. Misunderstandings, overreaction, provocation, and inappropriate emotional outbreaks are all easily triggered by trivial matters. Therefore, adolescents’ relationships with parents and siblings are likely to be unpleasant and even detached.

- “In the past, I chose to argue with my parents. I was very stubborn and stuck to my view, which might have worsened the relationship between my parents and me.”
- “As an adolescent during high school age, I always interpreted my parents’ behavior as purposefully and intentionally forcing their children, including me, to comply with their demands.”
- “As the youngest family member, I often have value conflicts with my mother. We try to challenge each other’s thoughts and values since neither wants to lose the battle.”

In this program, students acquired the ability to recognize the efforts made by their parents instead of blaming them without a second thought.

- “The attribution theory explains why my parents have irrational thoughts, so I can explain it to let them understand they should not have these thoughts. Also, this theory reminds me that I should not judge a person in a specific situation, and I should admit my faults when I am truly doing wrong.”
- “I understand that it is essential to listen to and respect others’ opinions. Therefore, I have started to listen to my parents’ words. One day, I asked my parents how they viewed some social issues, exchanging our views. I would also let my parents know my feelings and thoughts.”

By taking the perspective of family members, students were more patient, and tried to listen to their parents, and explain some issues to them, resulting in an enhanced mutual understanding that can help create a harmonized family environment. 

In addition, the family is harmonized by developing more empathy for parents and younger siblings. By advocating the strength perspective and a humanistic approach, students understood that it is crucial to be genuine and to actively listen, understand, and accept others.

- “Undeniably, the way my parents treated me with destructive language and physical abuse did cause childhood trauma. Yet, most importantly, I am now able to address these as challenges, motivations, and chances for change.”
- “Sometimes, I get angry with my younger brother and shout at him because he always bothers me when I am studying. After self-reflection with the use of the ABC model, I think although I don’t like it, actually, I can endure it and take it as a chance to have a short break from my study. Then I will no longer shout at him because of this, and it will make both him and me happier.”

Projecting these learning outcomes to family issues, students have acquired the capacity to facilitate positive and constructive family communication. Beyond that, some students indicated the sustainability of their learning, as they can utilize their knowledge to develop a supportive and warm family environment for their siblings and even future children.

- “I understand that we, humans, need unconditional love and acceptance to unearth our deepest potential; otherwise, it will lead to mental health problems or the thought that ‘I am worthless’. When I have a child, I need to remind myself to love and accept my child unconditionally, even if he or she fails to meet my expectations.”
- “I hope that my issues will be improved in the near future. I have a little brother who is nine years old. I would use the knowledge learned in this course to guide him toward better development.”

#### 4.2.6. Exploring the World

Some reflections in this domain were contributed by overseas students, who were more conscious of cultural differences, and able to make a worldwide comparison.

- “I am Burundian … The course covered how cultures can affect youth development, and it allowed me to realize that it is normal for my experience to be different from others.”
- “I am a Korean student who has experience studying in both my own and an overseas country. As the Korean education system typically demands a considerable amount of internet use, cyberbullying is common … From the lecture, I understand bullying in cyberspace has exceeded the rate of physical harassment in Korea and Hong Kong.”

With their learning experiences in Hong Kong, students spontaneously reflected on the differences between the two places, and compared them to expand their understanding, and destroy myths. For the local students, the course also motivated them to reflect and gain a more profound and expanded view of the world.

- “In the past, I just thought young people were the same everywhere when we were teens; the younger generations in different countries of the world are the same. But now I understand that is not true.”
- “Interestingly, the course offers a glimpse of adolescents’ issues that were entirely new to me … we should develop a broad-minded analysis in handling various social phenomena locally and globally.”

It is understandable that students reflect relatively less on exploring the world than in other domains, since it is hard to reflect on the world while situated within a classroom. The COVID-19 pandemic has limited students’ travel. However, this issue was re-examined in the individual essay, in which students were required to analyze youth issues worldwide from an academic perspective. This practice helped students to explore the world in an alternative way. Moreover, reflections on exploring the world were commonly linked to an appreciation of social diversity, which the quantitative results prove exists among students.

## 5. Discussion and Conclusions

Project Bridge is an innovative curriculum in the form of a positive education program that combines traditional Chinese virtues with Western theories with the purpose of building, enhancing, and promoting character and moral strengths. Based on both qualitative and quantitative data, we can confidently confirm its effectiveness in achieving this objective. The significant increases in self-reliance, responsibility bearing, and diversity appreciation are proven. As reflected in the qualitative data, there are certain degrees of enhancement in the six domains of the development journey. Students demonstrated significant growth in self-understanding of their development in the six character strength domains (cultivation of self, harmonizing the family, advocating group spirit, committing to professionalism, caring for society, exploring the world), which are aligned with positive education’s aim of nurturing individuals’ intrapersonal and interpersonal wellbeing [3,9]. Students experienced in-depth self-reflection upon their life experiences, and developed an enhanced understanding of self, seeing themselves from a broader perspective. Understanding their strengths and weaknesses, they attempted to make changes in their personality (i.e., self-cultivation) [7]. As such, their relationships with others were also improved. They adopted a broader perspective for understanding others, and found appropriate ways to interact with others so as to maintain good relationships (i.e., the wellbeing domain of positive relationships in positive education) [10,11]. For instance, they showed care and concern for others, such as friends and family (i.e., kindness, benevolence) [7,26,29], and even made breakthroughs to initiate relationships with others. The social competencies learnt were also applicable in handling relationships with people other than their closest circle, such as colleagues in work situations. Such an understanding of relationships even extended to a cross-cultural understanding of people and social issues. As such, the three practices (self-reliance, bearing responsibility, and appreciating diversity) were achieved, being indicators of flourishing/wellbeing [12], or “functioning well” [11] (p. 263) in the context of Chinese cultures [7,26].

On the other hand, students showed an understanding that the self is influenced by various social systems [41,42]. They not only learnt how to make adaptive changes to integrate into society, but also understood how society influenced them. They developed their own competencies from daily life experiences (e.g., the qualities required to become a teacher), and sought ways to actualize their own development. Such understanding facilitates the nurturance of a balanced self-development which can fit into the needs of both society and their own aspirations. The students demonstrated the quality of being communicants. All of the above indicates the project’s positive impact on students’ development of strength of character and morality. The positive outcomes of the project show the importance of contextualization in the implementation of positive education in order to nurture a sense of wellbeing in individuals that is adaptable to the culture of society [5,6]. As a brand-new approach to positive education that includes Chinese values and virtues in the Western-originated positive education framework, Project Bridge may be a cornerstone for the future trend of merging traditional Chinese ethics and virtues into positive education programs.

Higher education is important in facilitating students’ personal and professional development so that they can apply their knowledge and skills learnt in universities to manage their work and daily lives [69]. The positive outcomes of Project Bridge, in terms of the development of personal character strengths, interpersonal competencies, and broader perspectives featured by diversity and ethics of care, show that the integration between Chinese values and Western theories in positive education is feasible. Given the scarcity of evaluation of positive education programs with the inclusion of Chinese values to fit into the developmental needs of students in Chinese societies, this evaluative study shows the significance of providing empirical support for the development of university curricula for nurturing students’ all-round development in Chinese societies. Recognizing “the value of education in positioning itself as a regional and global hub of innovation” in Hong Kong [70] (p. 230), a practice implication is that it is essential to bear in mind the integration of Chinese values into Western-based theories in the design of positive education courses in universities. As such, students can achieve an all-round, balanced character; intellectual and moral development; and being equipped with a worldly mindset to cope with the challenges brought about by globalization independently, whilst being able to accommodate the local Chinese socio-cultural needs. Such an educational framework can also benefit international students, who can enhance their understanding of the Chinese values in order to better prepare themselves for pursuing smooth life and career development in Chinese societies.

In view of the positive outcomes of Project Bridge, this project can be extended in the future to secondary schools to promote the wellbeing of students in a broader manner. According to a meta-analysis conducted by Quinlan, Swain, and Vella-Brodrick [71], embracing character strengths in high school curricula improves students’ academic scores, social skills, and learning strengths, and reduces disordered behavior. Therefore, extending Project Bridge to secondary schools is considered to yield benefits for a larger population of adolescents. Since the curriculum of this project is designed for undergraduate students, programs for secondary school students need to be redesigned, modified, and redeveloped to fulfil the students’ developmental needs. In secondary school settings, the lecturing mode of transmitting knowledge should be converted into activity-based programs, which, though costly, can achieve better outcomes. On this note, it is recommended to conduct a small-scale trial to assess the effectiveness of the revised programs in selected schools before its full-fledged implementation. Given the lower reflective writing ability among secondary students, the use of a questionnaire survey with a pre- and post-test design is considered more appropriate for assessing learning outcomes. Measurement scales in this project can be adapted for secondary students’ levels. When the programs in selected secondary schools are mature enough and proven to be effective, the project can be extended to more schools.

## Figures and Tables

**Table 1:** Demographics of students across four semesters (N = 950).

Gender		
Male	318	33.47%
Female	632	66.53%
Study Year		
Year 1	283	29.79%
Year 2	344	36.21%
Year 3	123	12.95%
Year 4 or above	200	21.05%
Colleges and Schools		
College of Liberal Arts and Social Sciences	507	53.37%
College of Business	232	24.42%
College of Engineering	88	9.26%
School of Law	40	4.21%
School of Creative Media	26	2.74%
College of Science	22	2.32%
College of Vet Medicine & Life Sciences	23	2.42%
School of Data Science	5	0.53%
School of Energy and Environment	7	0.74%
Total	950	100%

**Table 2:** Cronbach’s alpha and chi-squared for each scale.

	Cronbach’s α	Chi-Squared Test		
		Value	df	Sig. (2-Tailed)
Self-esteem	0.858	29.353	20	0.081
Personal–social development, self-efficacy	0.968	89.318	88	0.441
Empowerment	0.784	39.253	36	0.326
Collective self-esteem	0.855	47.164	46	0.425
Emotional maturity	0.932	88.162	80	0.249
Personal responsibility	0.875	29.639	34	0.681
Openness to diversity	0.865	34.763	30	0.251
Moral development				
Conventional level stage 3	0.63	2.703	5	0.746
Conventional level stage 4	0.647	12.158	11	0.352
Postconventional level stages 5 and 6	0.706	19.627	16	0.237

**Table 3:** Quantitative evaluation results of Project Bridge (N = 950).

		Mean	S.D.	Sig. (2-Tailed)
Self-esteem	Pre-	2.541	0.411	0.000 ***
	Post-	2.733	0.409	
Personal–social development, self-efficacy	Pre-	4.125	0.612	0.000 ***
	Post-	4.573	0.677	
Empowerment	Pre-	2.505	0.387	0.000 ***
	Post-	2.832	0.307	
Collective self-esteem	Pre-	4.609	0.689	0.002 **
	Post-	4.687	0.629	
Emotional maturity	Pre-	2.777	0.386	0.016 *
	Post-	2.811	0.415	
Personal responsibility	Pre-	2.967	0.326	0.000 ***
	Post-	3.163	0.349	
Openness to diversity	Pre-	3.522	0.575	0.000 ***
	Post-	4.057	0.541	
Moral development				
Conventional level stage 3	Pre-	4.097	0.616	0.149
	Post-	4.059	0.602	
Conventional level stage 4	Pre-	3.761	0.504	0.846
	Post-	3.757	0.532	
Conventional level stage 5 and 6	Pre-	3.374	0.460	0.000 ***
	Post-	3.844	0.482	

Note: * *p* < 0.05. ** *p* < 0.01. *** *p* < 0.001.

**Table 4:** Quotations selected from students’ reflections.

	Number of Quotations
Cultivate self	516
Harmonize the family	99
Advocate group spirit	220
Commit to professionalism	116
Care for society	355
Explore the world	57
Total	1363

## Data Availability

The data that support the findings of this study are available on request from the corresponding author, T. Wing Lo. The data are not publicly available because they contain information that could compromise the privacy of the participants.
